# Clinical significance of incidental [18 F]FDG uptake in the gastrointestinal tract on PET/CT imaging: a retrospective cohort study

**DOI:** 10.1186/s12876-016-0545-x

**Published:** 2016-10-06

**Authors:** Eugenia Shmidt, Vandana Nehra, Val Lowe, Amy S. Oxentenko

**Affiliations:** 1 Division of Gastroenterology, Department of Internal Medicine, Mount Sinai Medical Center, New York, NY USA; 2 Division of Gastroenterology and Hepatology, Department of Internal Medicine, Mayo Clinic, Rochester, MN USA; 3 Division of Nuclear Medicine, Department of Internal Medicine, Mayo Clinic, Rochester, MN USA

**Keywords:** 18 F-fluorodeoxyglucose ([18 F]FDG), Positron emission tomography/computed, Tomography (PET-CT), Incidental colorectal lesions

## Abstract

**Background:**

The frequency and clinically important characteristics of incidental (18)F-fluorodeoxyglucose ([18 F]FDG) positron emission tomography (PET) uptake in the gastrointestinal tract (GIT) on PET/CT imaging in adults remain elusive.

**Methods:**

All PET/CT reports from 1/1/2000 to 12/31/2009 at a single tertiary referral center were reviewed; clinical information was obtained from cases with incidental (18)F-FDG uptake in the GIT, with follow-up through October, 2012.

**Results:**

Of the 41,538 PET/CT scans performed during the study period, 303 (0.7 %) had incidental GIT uptake. The most common indication for the PET/CT order was cancer staging (226 cases, 75 %), with 74 % for solid and 26 % for hematologic malignancies. Of those with solid malignancy, only 51 (17 %) had known metastatic disease. The most common site of GIT uptake was the colon, and of the 240 cases with colonic uptake, the most common areas of uptake were cecum (*n* = 65), sigmoid (*n* = 60), and ascending colon (*n* = 50). Investigations were pursued for the GIT uptake in 147 cases (49 %), whereas 51 % did not undergo additional studies, largely due to advanced disease. There were 73 premalignant colonic lesions diagnosed in 56 cases (tubular adenoma, *n* = 36; tubulovillous adenoma with low grade dysplasia, *n* = 27; sessile serrated adenoma, *n* = 4; tubulovillous adenoma with high grade dysplasia, *n* = 3; villous adenoma, *n* = 3), and 20 cases with newly diagnosed primary colon cancer. All 20 (100 %) patients with malignant colonic lesions had a focal pattern of [18 F]FDG uptake. Among cases with a known pattern of [18 F]FDG uptake, 98 % of those with premalignant lesions had focal [18 F]FDG uptake. Eighteen (90 %) of the cases with newly diagnosed colon cancer were not known to have metastatic disease of their primary tumor. Areas of incidental uptake in the ascending colon had the greatest chance (42 %) of being malignant and premalignant lesions than in any other area.

**Conclusion:**

Focality of uptake is highly sensitive for malignant and premalignant lesions of the GIT. In patients without metastatic disease, incidental focal [18]FDG uptake in the GIT on PET/CT imaging warrants further evaluation.

## Background

Positron emission tomography (PET) is a nuclear imaging technique that has been widely used in oncology for the detection of neoplasia [1]. In 1998, the Centers for Medicare and Medicaid Services (CMS) approved coverage for PET imaging in the evaluation of indeterminate solitary pulmonary nodules and initial staging of lung cancer. Since that time, CMS has expanded its coverage for various investigations of different types of cancers including breast, lung, colorectal, esophageal, head and neck, lymphoma and melanoma [2]. The use of PET imaging continues to increase, and is now standard in oncologic practice.

Fluorine-18 fluorodeoxyglucose ([18 F]FDG) is the most commonly used radiolabeled tracer in PET scans. [18 F]FDG is taken up intracellularly by various tissues and accumulate during glucose metabolism, which occurs at a relatively higher rate in cancer cells. However, cancer cells are not the only cells that are metabolically hyperactive. Inflammation, infection and other non-neoplastic conditions such as hyperplastic colorectal polyps can lead to increased 18 F- FDG accumulation [3]. For this reason, PET scans have a high sensitivity but a low specificity for colorectal cancer [3, 4].

PET/computed tomography (CT) scanning offers two technologies that combine metabolic abnormality detection with anatomic localization [5, 6]. Compared to PET alone, this combination has been shown to be superior in localizing lesions and differentiating between physiologic and malignant uptake of [18 F]FDG [6].

Given the use of PET/CT has become increasingly common in oncologic practice, it is not surprising that with increased use comes incidental [18 F]FDG uptake that was not anticipated, forcing the ordering physician to decide how to address such findings. Numerous studies explored incidental [18 F]FDG uptake in the GIT and reported rates ranging from 0.5 % to 2.6 % [7–14]. Studies have shown that a focal pattern of incidental [18 F]FDG uptake in the GIT is more likely to be malignant than a non-focal pattern [7, 15]. However, these studies are limited by relatively small numbers.

The purpose of our study was to determine the frequency, clinical characteristics and patterns of [18 F]FDG uptake in lesions found incidentally in the GIT on PET/CT imaging in adults.

## Methods

We searched the Mayo Clinic electronic record database from 1/1/2000 to 12/31/2009 for patients greater than 18 years of age who had the terms “bowel”, “colonoscopy” and “intestine” in their whole body PET/CT reports. All of these radiology reports were reviewed by a single investigator who identified cases with incidental uptake on PET/CT imaging. An incidental finding was defined as an area of [18 F]FDG uptake in the GIT that could not be expected based on the patient’s known medical history at the time of the PET/CT scan. Patients whose indication for PET/CT imaging was gastrointestinal cancer were excluded. The medical records were reviewed by a single investigator for demographic information; medical history; indication for PET/CT imaging; location and pattern of [18 F]FDG tracer uptake; date of most recent previous PET/CT study, if applicable; work-up launched to investigate the incidental finding, if any; new diagnoses from subsequent investigational studies. Follow-up was performed through October 2012. The study was approved by the Mayo Clinic Institutional Review Board.

## Results

**Fig. 1:**
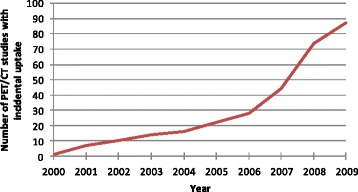
Number of PET/CT studies with incidental FDG uptake by year, 2000–2009

Among the 41,538 PET/CT scans performed during the study period, 303 (0.7 %) scans from 288 unique patients had incidental GIT uptake. The incidence of incidental [18 F]FDG uptake in the GIT on PET/CT scans increased substantially during the study period (Fig. 1). The mean age for all patients, patients with malignant lesions and patients with premalignant lesions was 66.9 years (standard deviation 11.8), 71.1 years (standard deviation 11.6) and 68.5 years (standard deviation 12.0), respectively.

Two hundred and forty-one scans reported a pattern of uptake, with 232 with a focal pattern, eight with a diffuse pattern and one with both diffuse and focal components. A diffuse pattern of uptake was noted in multiple colonic locations (one case), sigmoid and rectum (one case), rectum only (one case), small bowel (one case), entire colon (one case), ascending colon (one case), not specified (two cases). the 303 cases, 147 (49 %) underwent further investigations. The remaining 51 % did not undergo further investigations largely due to advanced cancer. Studies pursued were colonoscopy (108 cases), extended or routine esophagogastroduodenoscopy (EGD) (seven cases), CT abdomen (four cases), CT enterography (three cases), balloon-assisted endoscopy (one case), capsule study (one case), flexible sigmoidoscopy (one case), CT colonography (one case), follow-up PET scan (three cases), laparoscopy (one case), and multiple investigations (seventeen cases). In nineteen other cases, endoscopy (colonoscopy in eighteen cases and sigmoidoscopy in one case) was recommended, but results were unavailable because these studies were either not performed or the patient was lost to follow-up. Among all 303 cases, the most common indication for the PET/CT order was cancer staging (226 cases, 75 %), with 74 % (167 cases) for solid and 26 % (59 cases) for hematologic malignancies; other indications included follow-up of an indeterminate nodule/mass (9 %, 28 cases) and paraneoplastic features (6 %, seventeen cases). Of those with solid malignancy, only 51 (17 %) had known metastatic disease.

**Fig. 2:**
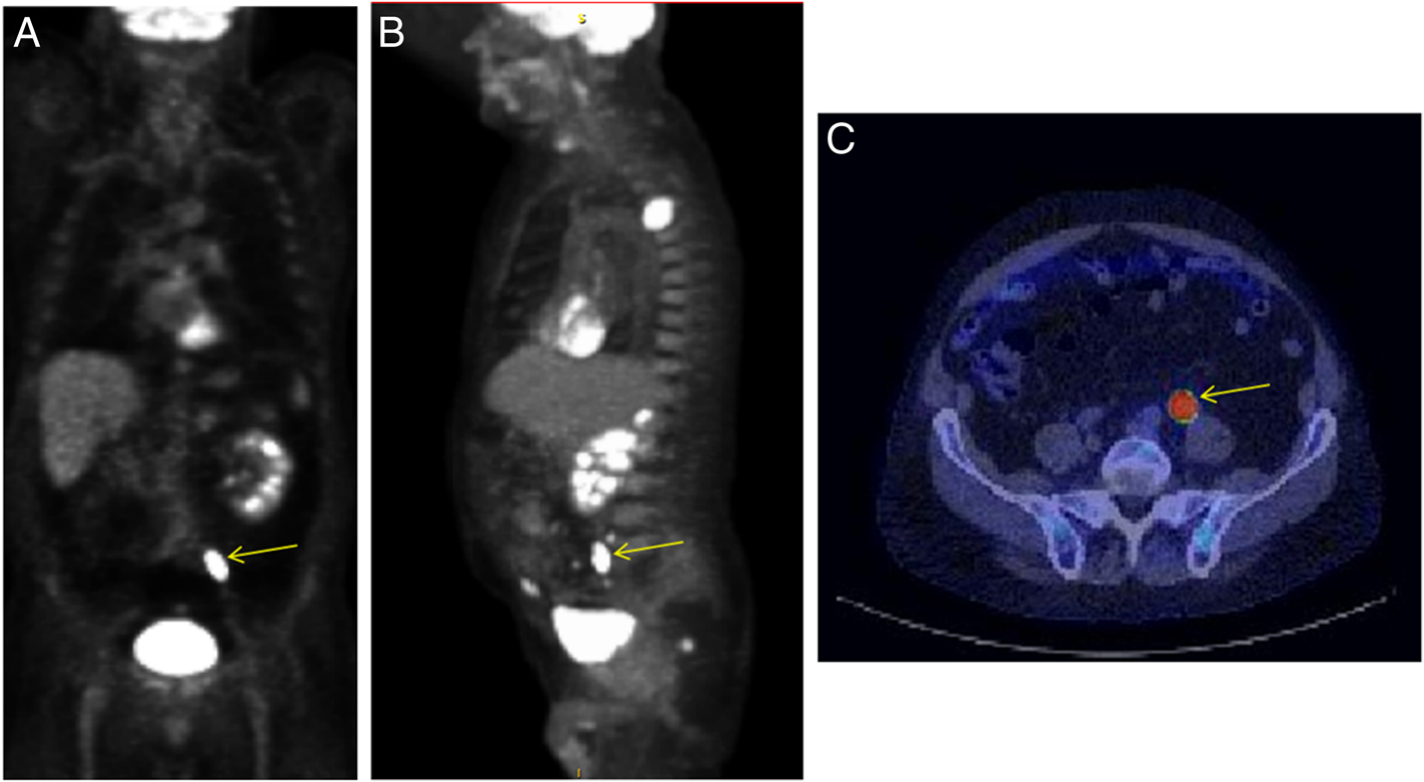
Focal [18 F]FDG uptake in a 79 year old male who was evaluated for an isolated right upper lobe pulmonary nodule. (**a**) coronal and (**b**) sagittal PET slices show indeterminate FDG avid foci (*arrows*) within the rectum. Area of increased uptake (*arrow*) was localized by PET/CT (**c**). Tissue biopsy revealed invasive moderately differentiated (grade 3 of 4) adenocarcinoma

Among all patients, patients with malignant lesions and patients with premalignant lesions, females comprised 44 % (126 females), 45 % (nine females) and 45 % (24 females), respectively. There were 20 malignant lesions identified in 20 patients, representing 14 % of all patients who underwent additional investigations. The malignant lesions were found in the cecum (25 % cases), ascending colon (25 % cases), transverse colon (20 % cases), descending colon (15 % cases) and rectum (15 % cases) (Table 1). Lesions in the ascending colon had the greatest likelihood of being malignant or pre-malignant; 42 % (21 cases) of all lesions in the ascending colon were identified as premalignant or malignant. Eighteen (90 %) of the cases with newly diagnosed colon cancer were not known to have metastatic disease of their primary tumor. For five cases, the T stage was not known because surgical resections were not performed at our institution. Of the remaining fifteen cases, ten were T3, three were T4 and one was T1. Six cases had nodal involvement and three had distant metastases. Figure 2 demonstrates a case in which a rectal adenocarcinoma was eventually diagnosed in a patient undergoing PET/CT scan for evaluation of a pulmonary nodule.

**Table 1:** Five most common colon locations of ^18^F-fluorodeoxyglucose ([18 F]FDG) uptake in patients who underwent colonoscopy^a^

Malignant lesions, no. (%)		Premalignant lesions, no. (%)		All, no. (%)	
Cecum	5 (25)	Cecum	16 (28)	Cecum	65 (21)
Ascending colon	5 (25)	Ascending colon	16 (28)	Sigmoid	60 (20)
Transverse colon	4 (20)	Sigmoid	13 (23)	Ascending colon	50 (17)
Descending colon	3 (15)	Transverse colon	6 (11)	Transverse colon	36 (12)
Rectum	3 (15)	Descending colon	4 (7)	Descending colon	29 (10)

^a^ = 20 areas of uptake in patients that were later found to have malignancies, 56 areas of uptake in patients that were later found to have premalignant lesions, 303 all areas of incidental uptake

There were 73 premalignant lesions identified in 56 PET/CT scans among 53 patients, representing 38 % areas of incidental [18 F]FDG uptake that underwent additional investigations. There were 36 tubular adenomas, 27 tubulovillous adenomas with low grade dysplasia, four sessile serrated adenomas, three tubulovillous adenomas with high grade dysplasia and three villous adenomas.

Among all PET/CT scans in our study, 55 (18 %) had areas of incidental uptake that were endoscopically examined and found to be non-neoplastic. Thirty-eight (69 %) of these scans were noted to have a focal pattern of [18 F]FDG uptake. Among these 55 scans, 39 (71 %) had no abnormalities identified.

Specifically outside the colorectum, [18 F]FDG uptake was noted in the esophagus (five cases), stomach (five cases), duodenum (four cases), jejunum (one case), ileum (nine cases) and other locations in the abdomen that were not specifically ascribed to a particular organ (sixteen cases). Endoscopic work up revealed the following pathology in these cases: erosive esophagitis (two cases), tubulovillous adenoma in the duodenum (one case), hyperplastic polyp in the gastric cardia (one case), antral erosion (one case), celiac disease (one case), Barrett’s esophagus (one case), and NSAID-induced ileitis (one case).

Of the eight areas with a diffuse pattern of uptake in the bowel, five were endoscopically examined and one preneoplastic lesion (tubular adenoma) was identified in the sigmoid. In two additional cases, further work up revealed small bowel intussusception (one case) and celiac disease (one case). Of the five remaining cases with an unknown etiology for the [18 F]FDG uptake, one patient was noted to be on metformin (dose was 500 mg twice daily). There were three polyps of unknown histology because one was not biopsied and two were obtained at an outside hospital.

Table 2 demonstrates patterns of [18 F]FDG uptake in areas that turned out to be malignant, premalignant and non-neoplastic. The sigmoid colon was the most common site of incidental [18 F]FDG uptake that corresponded to no known pathology. Among cases of known pattern of [18 F]FDG uptake, a focal pattern was seen in 100 %, 98 % and 88 % of colonic lesions that turned out to be malignant, pre-malignant and non-neoplastic, respectively.

**Table 2:** Patterns of [18 F]FDG uptake among malignant, premalignant and non-neoplastic lesions

	Malignant lesions, no. (%)	Premalignant lesions, no. (%)	Non-neoplastic lesions per endoscopy^a,b^, no. (%)
Focal uptake, no. (% of cases with known pattern of uptake)	12 (100)	46 (98)	38^b^ (88)
Diffuse uptake, no (% of cases with known pattern of uptake)	0 (0)	1 (2)	4 (9)

^a^ = 54 areas of uptake

^b^ = 12 patients had an unknown pattern of uptake

## Discussion

Due to the wide use of PET/CT imaging in oncology, incidental [18 F]FDG uptake in the GIT is found frequently. In our study, incidental [18 F]FDG uptake in the GIT was found in 0.7 % patients who had PET/CT imaging. Treglia and colleagues reported a 1.1 % prevalence of a focal pattern of colorectal uptake of [18 F]FDG [8]. Peng and colleagues reported a similar prevalence of 1.35 % [9]. In the present study, 0.6 % incidental [18 F]FDG uptake had a focal pattern of [18 F]FDG uptake. Possible reasons for this disparity in reported prevalence may be differences in patient populations and practice settings. Compared to other studies, the present study is the largest, spanning 10 years and more than 41,000 whole-body [18 F]FDG PET scans.

Our findings underscore previously reported data that diffuse [18 F]FDG uptake is not suggestive of a neoplastic lesion. None of the malignant lesions in our study had a diffuse pattern of uptake. Only one patient had a diffuse pattern of [18 F]FDG uptake on a PET scan and an eventual diagnosis of a premalignant lesion; however, the area of the premalignant lesion was different from the area of the [18 F]FDG uptake. This patient had celiac disease and a diffuse pattern of [18 F]FDG uptake in the small bowel, cecum, left colon and sigmoid colon. A colonoscopy was performed two months later to further investigate findings of the abnormal PET scan and revealed a tubular adenoma in the ascending colon. In 2002, Tatlidil and colleagues examined 27 patients with incidental PET uptake in the gastrointestinal tract (GIT) who subsequently underwent endoscopy [7]. The authors concluded that nodular high [18 F]FDG uptake should be investigated with colonoscopy, a diffuse pattern of uptake is likely to be normal and segmental high uptake is suggestive of inflammation [7]. The present study offers a significantly larger patient population to support the same conclusion regarding a diffuse pattern of [18 F]FDG uptake.

Among the 147 cases of incidental [18 F]FDG uptake who underwent further investigations, more than half (52 %) had colorectal pathology in the form of malignant or premalignant lesions. This is similar to the frequency reported by the study reported by Treglia and colleagues that found 64 % of patients who had further investigations were diagnosed with malignant or premalignant lesions [8]. Given the high likelihood that an area of incidental [18 F]FDG uptake in the GIT may represent a malignant or premalignant lesion, our data supports previously stated recommendations to perform a colonoscopy for further investigation.

Among cases in which the pattern of [18 F]FDG uptake was known, 40 % had focal 18 F- FDG uptake in areas that were later found to be endoscopically normal. Possible etiologies are active smooth muscle, metabolically active mucosa, swallowed secretions, or colonic microbial uptake.

The cecum and ascending colon were the most common areas of pathology; each location had 21 areas of incidental [18 F]FDG uptake that turned out to be malignant or premalignant. Areas of incidental uptake in the ascending colon had the greatest chance (42 %) of being malignant and premalignant lesions than in any other area. These findings are different from those reported by others, in which the greatest proportion of malignant and premalignant lesions was found in the distal colon and rectum [8, 9].

The sigmoid colon was the most common site of incidental [18 F]FDG uptake that corresponded to no known pathology, per endoscopy. Treglia and colleagues reported similar findings with the largest number of non-neoplastic lesions in the sigmoid colon [8]. However, other reports describe an increased false-positive rate of [18 F]FDG uptake in the ascending colon [9, 16, 17]. A possible explanation for increased uptake in the ascending colon could be diarrhea, since this has been shown to cause focal [18 F]FDG uptake on PET scans [18]. Other investigators proposed that a higher uptake within the ascending colon and cecum may be secondary to a higher concentration of lymphocytes in the region [16]. The high false positive rate observed in the sigmoid colon in the present study remains without explanation, although given diverticular disease is most common in the sigmoid colon, one has to wonder if a mildly inflamed diverticulum could account for this.

Our study has several limitations. The study is retrospective and is therefore vulnerable to sampling bias. Additionally, half of cases with incidental [18 F]FDG uptake were not further investigated and the pathology of those lesions remains unknown in this retrospective cohort. Therefore, the evidence in this study may not be sufficient to provide an accurate prevalence of premalignant and malignant lesions in patients with incidental [18 F]FDG uptake on PET/CT imaging. Our data is limited to a single, tertiary referral center and our patient population may not be representative of the general patient population at non-tertiary referral centers; however, given it is the largest sample for which incidental GIT uptake is reported, it is likely more representative compared to smaller multicenter studies.

## Conclusion

Focal [18]FDG uptake in the GIT on PET/CT imaging is highly sensitive for malignant and premalignant lesions of the GIT. In patients without metastatic disease, incidental focal [18]FDG uptake in the GIT on PET/CT imaging warrants further evaluation.

## Abbreviations

[18 F]FDG: (18)F-fluorodeoxyglucose
CMS: Centers for medicare and medicaid services
CT: Computed tomography
GIT: Gastrointestinal tract
PET: Positron emission tomography
